# Plaque volume and plaque risk profile in diabetic vs. non-diabetic patients undergoing lipid-lowering therapy: a study based on 3D intravascular ultrasound and virtual histology

**DOI:** 10.1186/s12933-017-0637-0

**Published:** 2017-12-07

**Authors:** Tomas Kovarnik, Zhi Chen, Gary S. Mintz, Andreas Wahle, Kristyna Bayerova, Ales Kral, Martin Chval, Karel Kopriva, John Lopez, Milan Sonka, Ales Linhart

**Affiliations:** 1 2nd Department of Medicine-Department of Cardiovascular Medicine, First Faculty of Medicine, Charles University in Prague and General University Hospital in Prague, II. interni klinika VFN a 1. LF UK, U nemocnice 2, 128 08 Praha 2, Czech Republic; 2 Department of Electrical & Computer Engineering and Iowa Institute for Biomedical Imaging, The University of Iowa, Iowa City, IA USA; 3 Cardiovascular Research Foundation, New York, USA; 4 Institute for Research and Development of Education, Faculty of Education, Charles University in Prague, Prague, Czech Republic; 5 Cardiology Department, Na Homolce Hospital, Prague, Czech Republic; 6 Loyola University Stritch School of Medicine, Maywood, IL USA

**Keywords:** Intravascular ultrasound, Diabetes mellitus, Atherosclerosis, Statins

## Abstract

**Background:**

Coronary atherosclerosis progresses faster in patients with diabetes mellitus (DM) and causes higher morbidity and mortality in such patients compared to non-diabetics ones (non-DM). We quantify changes in plaque volume and plaque phenotype during lipid-lowering therapy in DM versus non-DM patients using advanced intracoronary imaging.

**Methods:**

We analyzed data from 61 patients with stable angina pectoris included to the PREDICT trial searching for prediction of plaque changes during intensive lipid-lowering therapy (40 mg rosuvastatin daily). Geometrically correct, fully 3-D representation of the vascular wall surfaces and intravascular ultrasound virtual histology (IVUS-VH) defined tissue characterization was obtained via fusion of two-plane angiography and IVUS-VH. Frame-based indices of plaque morphology and virtual histology analyses were computed and averaged in 5 mm long baseline/follow-up registered vessel segments covering the entire length of the two sequential pullbacks (baseline, 1-year). We analyzed 698 5-mm-long segments and calculated the Liverpool active plaque score (LAPS).

**Results:**

Despite reaching similar levels of LDL cholesterol (DM 2.12 ± 0.91 mmol/l, non-DM 1.8 ± 0.66 mmol/l, p = 0.21), DM patients experienced, compared to non-DM ones, higher progression of mean plaque area (0.47 ± 1.15 mm^2^ vs. 0.21 ± 0.97, p = 0.001), percent atheroma volume (0.7 ± 2.8% vs. − 1.4 ± 2.5%, p = 0.007), increase of LAPS (0.23 ± 1.66 vs. 0.13 ± 1.79, p = 0.018), and exhibited more locations with TCFA (*Thin-Cap Fibro-Atheroma*) plaque phenotype in 5 mm vessel segments (20.3% vs. 12.5%, p = 0.01). However, only non-DM patients reached significant decrease of LDL cholesterol. Plaque changes were more pronounced in PIT (*pathologic intimal thickening*) compared to TCFA with increased plaque area in both phenotypes in DM patients.

**Conclusion:**

Based on detailed 3D analysis, we found advanced plaque phenotype and further atherosclerosis progression in DM patients despite the same reached levels of LDLc as in non-DM patients.

*Trial registration* ClinicalTrials.gov identifier: NCT01773512

## Background

Studies with intravascular ultrasound (IVUS) have shown that atherosclerosis progression can be stopped [1, 2] or reversed [3–5] by using aggressive lipid-lowering therapy. However, these changes are less pronounced in diabetic patients compared to non-diabetic patients despite the same reduction of LDL cholesterol (LDLc) [6, 7]. Furthermore, poor glycemic control in diabetic patients is associated with plaque progression [8, 9] and the presence of diabetes is found as a predictor of plaque progression despite achieving very low levels of LDLc [10].

Atherosclerosis occurs earlier in diabetic patients [11] and shortens their life expectancy [12]. Impaired glycemic homeostasis has a direct influence on the formation and propagation of atherosclerotic plaque [13], and diabetic patients are at risk for a first myocardial infarction that is comparable to non-diabetic patients who have already experienced at least one myocardial infarction [14]. As a consequence, diabetic patients with coronary artery disease have a higher morbidity and mortality compared to non-diabetics [15].

Plaque composition is an important factor related to future clinical presentation [16]. Using IVUS-virtual histology (IVUS-VH), six plaque phenotypes can be distinguished corresponding to those described in the American Heart Association’s Committee on Vascular Lesions [17]. These phenotypes are as follows:

- no lesion—NL (plaque burden less than 40%)
- pathologic intimal thickening—PIT
- fibrous plaque—FP
- fibro-calcified plaque—FcP
- thick cap fibro-atheroma—ThCFA
- thin cap fibro-atheroma—TCFA.

Fibro-atheromas (ThCFA and TCFA) are risk factors for future cardiac events (TCFA more so than ThCFA) [18, 19]. The aim of this study was to compare changes in plaque phenotype during lipid-lowering therapy using 3D reconstruction of coronary arteries based on fusion of IVUS-VH with coronary angiography in diabetic patients (DM group) versus patients without diabetes mellitus (non-DM group). Image data were obtained at baseline and at 1-year follow-up, covering the entire length of IVUS-VH pullback and allowing us to follow changes of plaque phenotypes in a systematic, representative fashion. Unlike the frame-based analysis that is the most common approach in similar trials [1–5], we divided the imaged vessels into 5 mm segments after geometrically correct, 3-D vessel reconstruction via fusion of two-plane angiography and IVUS-VH.

## Methods

### Study population, catheterization and IVUS imaging

Patient data were taken from the database of the PREDICT trial assessing the ability to predict plaque behavior during intensive lipid-lowering therapy (rosuvastatin 40 mg daily) (ClinicalTrials.gov identifier: NCT01773512). DM-patients were identified as those receiving treatment with either oral antidiabetics or insulin and also based on patient’s history of DM treated by diet. There were no patients with DM type 1 in the study. All patients signed an informed consent, and the study was approved by the local ethics committee.

In all cases only one coronary artery was examined per patient. From the acquired data, only those patients who fulfilled all the following criteria were enrolled:

1. IVUS-VH of a native coronary artery with stenosis ≤ 50% of lumen diameter determined by angiography with no indication for either percutaneous coronary intervention (PCI) or coronary artery bypass grafting (CABG).
2. Good-quality baseline and follow up IVUS-VH pullbacks (i.e., without noticeable pullback speed discontinuity).
3. Imaged vessels free of severe calcification to avoid inconsistency of IVUS-VH plaque type determination in areas of acoustic shadowing.
4. Baseline and follow up pullbacks that were least 30 mm long and that had at least 25 mm long segments that were imaged both at baseline and at follow-up.

One segment from each patient was chosen for the study. The lesion located in the proximal coronary segment or located in a non-angulated segment was selected in cases when several similar stenoses were present in the imaged vessel.

IVUS was performed according to the standard protocol using a phased-array IVUS probe (Eagle Eye 20 MHz 2,9F monorail, Volcano Corporation, Rancho Cordova, California), with automatic pullback at 0.5 mm/s (research pullback, model R-l00, Volcano Corporation, Rancho Cordova, California). After administration of 200 μg of intracoronary nitroglycerin, the IVUS catheter was inserted into the target vessel beyond a distal fiduciary point, and then pulled back to the aorto-ostial junction. The proximal fiduciary point was either the left main bifurcation in the left coronary artery or the first branch or well-defined calcification in the right coronary artery. The distal fiduciary point was determined by the presence of a reproducible side branch.

Original B-mode IVUS pullback image data were acquired at the *Charles University Hospital in Prague*, *Czech Republic,* archived onto DVDs, and transferred to the *Iowa Institute for Biomedical Imaging, The University of Iowa, Iowa City, Iowa, USA* for quantitative analysis. For each IVUS frame, luminal and external elastic membrane (EEM) surfaces were automatically segmented using fully three-dimensional LOGISMOS graph-based approach [20, 21]. This system has been developed at The University of Iowa for simultaneous, optimality-guaranteeing segmentation of multiple mutually-interacting surfaces and 3D/4D analysis of serial IVUS images of coronary atherosclerosis.

**Fig. 1:**
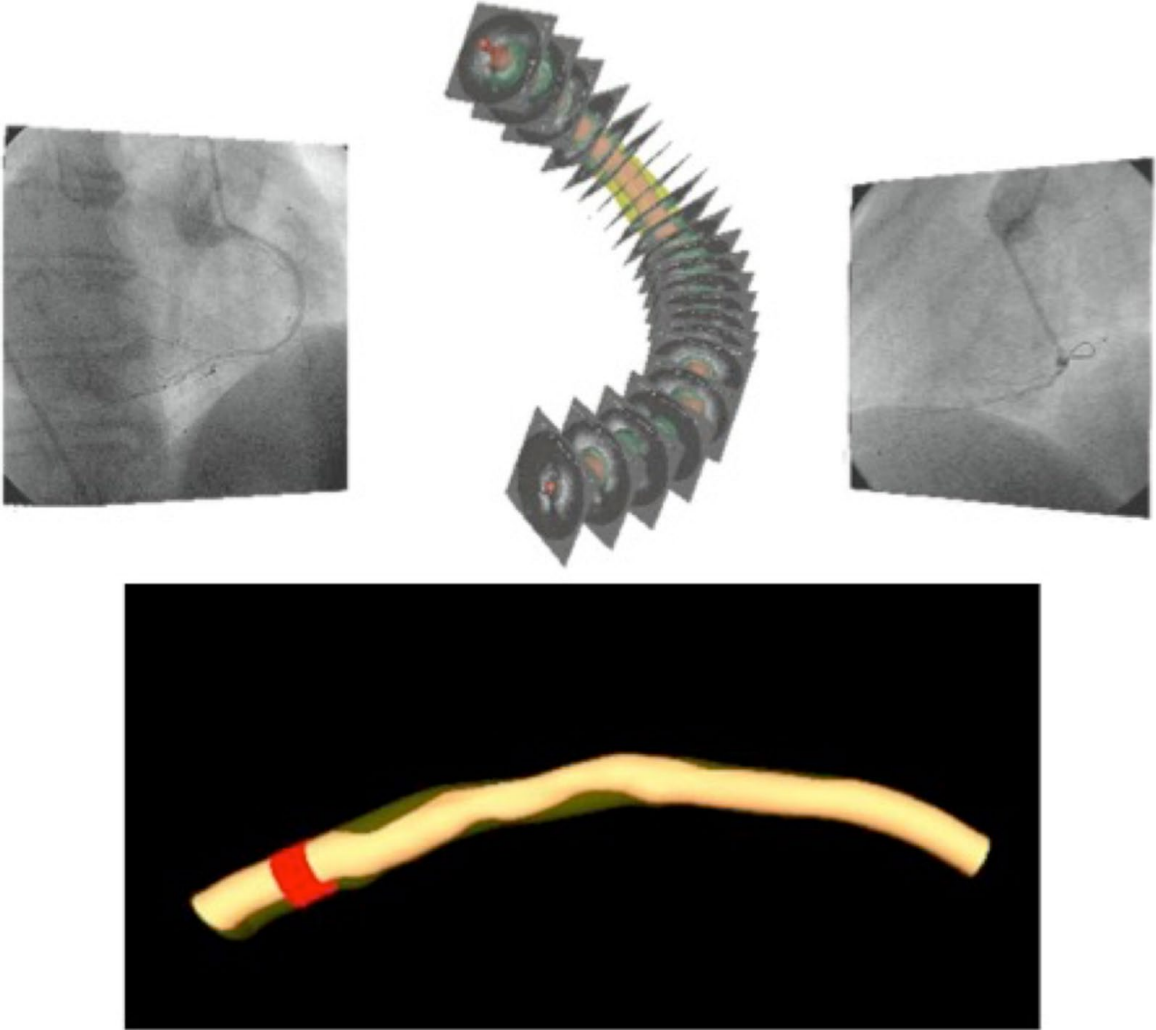
3D model of coronary artery done by fusion of CAG and IVUS and marked 5 mm vessel segment

Automatically determined surfaces were reviewed and algorithmically refined by one expert cardiologist (TK) using an operator-guided computer-aided interface [22]. EEM and lumen surfaces/contours served as the input for off-line IVUS-VH computation using Volcano’s research software that is identical to that available on the IVUS console, but allows VH computations based on user-supplied segmentation (Volcano Corp.). Employing our previously reported approach [23], a geometrically correct, full 3-D representation of the vascular wall surfaces and IVUS-VH-defined tissue characterization was obtained via fusion of two-plane angiography and IVUS-VH. This geometrically correct 3-D model served as a basis for quantitative morphologic analyses and quantitative assessment of plaque composition in every frame of the imaged vessel [24]. Using this approach, vessel models were obtained for both the baseline and follow-up pullbacks. After identification of corresponding vascular landmarks in the two 3-D vessel models, the patient-specific model pairs were co-registered. Frame-based indices of plaque morphology and IVUS-VH analyses were computed and averaged in 5 mm long baseline/follow-up registered vessel segments (Fig. 1).

Vessel and plaque measurement morphologic indices included:

- external elastic membrane (EEM) cross-sectional area (CSA),
- lumen CSA,
- percent atheroma volume (PAV) calculated as: PAV = 100 × Σ (EEM_area_ − Lumen_area_)/Σ EEM_area_, where EEM_area_ is the cross-sectional area of the external elastic membrane and Lumen_area_ is the cross-sectional area of the lumen.

### Phenotype definitions

**Fig. 2:**
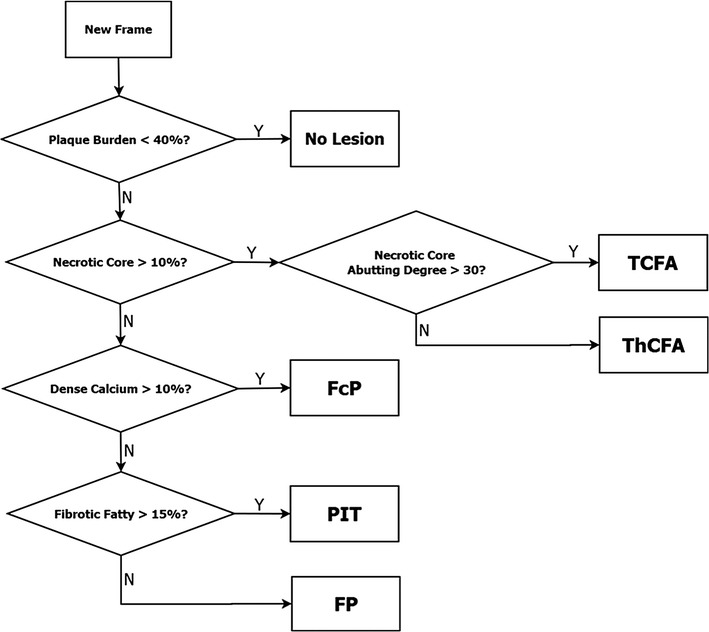
Determination of plaque phenotype in frame-based analysis

IVUS-VH data classifies plaque into four components: fibrous (F), fibro-fatty (FF), dense calcification (DC), and necrotic core (NC). Phenotypes of all 5 mm-long vessel segments were classified into six categories (NL, FcP, FP, PIT, ThCFA, and TCFA) according to the flowchart given in Fig. 2 and as previously published [25]. Each vessel segment was labeled according to the most advanced plaque phenotype found in each frame (in the following ascending order from least to most advanced phenotype: NL, PIT, FP, FcP, ThCFA, TCFA).

In addition, we calculated the *Liverpool Active Plaque Score* (LAPS) as adapted from Murray et al. [26]: − 2.149 + 0.68 × NC/DC + 3.39 × MLA + 5.1 (if remodeling index was > 1.05) + 3.7 × VH-TCFA. LAPS was calculated for each frame. LAPS for 5 mm vessel segments was the highest LAPS found in this segment, and LAPS for baseline/follow-up examination was the mean risk score from all analyzed 5 mm vessel segments.

### Statistical analysis

Mean values ± standard deviations (or percentages) were calculated for all numerical variables. Differences of two numerical datasets were examined by Student’s *t* test. Mann–Whitney *U* test was used instead if the sample could not be assumed to be normally distributed. For categorical variables like diabetes status, contingency tables were used to display frequency distributions. Statistical significance was subsequently calculated by Fisher’s exact test. To investigate segmental plaque morphological changes, mixed-effect analysis with “patient” as random effect is used to correct the clustering of multiple segments within patients. The R statistical-computing environment was employed for analysis. A *p*-value of 0.05 denoted statistical significance.

## Results

The analysis was performed in 698 5-mm-long vessel segments from 61 patients. Total number of DM patients was 17 and they were treated as follows: 2 patients with insulin, 3 patients with diet only, 12 patients with metformin. Patient demographic information is presented in Table 1. Analyzed vessel sections were 70.5 ± 15.8 mm long on average in the DM group and 72.9 ± 18.6 mm long in the non-DM group (p = 0.63). Changes of plaque volumes are summarized in Table 2, and changes in plaque composition in Table 3. Liverpool active risk score and its changes are summarized in Table 4.

**Table 1:** Patient demographics

	DM (17 pts.)	Non-DM (44 pts.)	p
Age (years)	62.2 ± 7.1	62.0 ± 10.9	0.96
Men	12 (70.6%)	35 (79.5%)	0.51
Arterial hypertension	17 (100%)	39 (88.6%)	0.31
Current smoking	4 (23.5%)	15 (34.1%)	0.54
MI in past	7 (50%)	20 (54.1%)	0.8
Statin naive	3 (17.6%)	10 (22.7%)	0.98
Beta blockers	16 (94.1%)	28 (63.6%)	0.02
ACEI	15 (88.2%)	36 (81.8%)	0.71
Baseline Ch (mmol/l)	4.21 ± 0.75	4.31 ± 1.22	0.69
Follow-up Ch (mmol/l)	3.79 ± 0.99	3.35 ± 0.99	0.13
Change of Ch (mmol/l)	− 0.42 ± 0.89	− 0.97 ± 1.45	0.09
Baseline LDLc (mmol/l)	2.42 ± 0.53	2.46 ± 1.04	0.86
Follow-up LDLc (mmol/l)	2.12 ± 0.91	1.8 ± 0.66	0.21
Change of LDLc (mmol/l)	− 0.3 ± 0.52	− 0.66 ± 1.09	0.18
Baseline HDLc (mmol/l)	1.08 ± 0.38	1.23 ± 0.3	0.14
Follow-up HDLc (mmol/l)	1.09 ± 0.38	1.23 ± 0.27	0.19
Change of HDLc (mmol/l)	0.02 ± 0.52	− 0.01 ± 0.24	0.88
Baseline TAG (mmol/l)	1.59 ± 0.74	1.50 ± 0.88	0.4
Follow-up TAG (mmol/l)	1.52 ± 0.95	1.21 ± 0.52	0.22
Change TAG (mmol/l)	− 0.07 ± 0.96	− 0.29 ± 0.84	0.42
apo A (mmol/l)	0.08 ± 0.15	1.36 ± 0.32	0.16
apo B (mmol/l)	− 0.08 ± 0.22	− 0.13 ± 0.33	0.54
Baseline hs-CRP (mg/l)	2.28 ± 2.53	3.14 ± 4.31	0.49
Follow-up hs-CRP (mg/l)	4.87 ± 5.5	3.43 ± 4.0	0.3
Change of hs-CRP (mg/l)	2.6 ± 4.24	0.29 ± 5.1	0.13
Baseline glycemia (mmol/l)	7.04 ± 2.41	5.59 ± 0.78	0.0009
Follow-up glycemia (mmol/l)	7.51 ± 2.1	5.67 ± 0.78	< 0.0001
Change of glycemia (mmol/l)	0.04 ± 2.83	− 0.18 ± 1.45	0.69

*MI* myocardial infarction, *ACEI* angiotensin-converting enzyme inhibitors, *Ch* cholesterol, *LDLc* low density lipoprotein cholesterol, *HDLc* high density lipoprotein cholesterol, *TAG* triacyl glyceroles, *apo A* apolipoprotein A, *apo B* apolipoprotien B, *hs-CRP* high sensitive C reactive protein

**Table 2:** IVUS analysis of plaque morphology expressed as mean values per 5 mm vessel segments

	DM (17 pts, 192 segments)	Non-DM (44 pts, 506 segments)	p
Lumen area BL (mm^2^)	8.53 ± 3.8	9.27 ± 3.89	0.44
Lumen area FU (mm^2^)	8.54 ± 3.81	9.19 ± 3.79	0.53
Δ Lumen area (mm^2^)	0.02 ± 1.1	− 0.08 ± 1.2	0.54
p value for change between BL and FU	0.96	0.64	
Vessel area BL (mm^2^)	15.79 ± 5.67	16.66 ± 5.62	0.5
Vessel area FU (mm^2^)	16.01 ± 5.7	16.1 ± 5.37	0.94
Δ Vessel area (mm^2^)	0.22 ± 1.4	− 0.56 ± 1.8	*0.04*
p value for change between BL and FU	0.54	*0.01*	
Plaque area BL (mm^2^)	7.26 ± 2.99	7.39 ± 3.31	0.7
Plaque area FU (mm^2^)	7.47 ± 3.08	6.91 ± 3.08	0.42
Δ Plaque area (mm^2^)	0.21 ± 0.97	− 0.47 ± 1.15	*0.001*
p value for change between BL and FU	0.34	*0.001*	
Plaque atheroma volume BL	45.7 ± 5.0%	44.6 ± 8.0%	0.60
Plaque atheroma volume FU	46.4 ± 4.2%	43.2 ± 7.8%	0.14
Δ Plaque atheroma volume	0.7 ± 2.8%	− 1.4 ± 2.5%	*0.007*
p value for change between BL and FU	0.56	0.4	

Plaque burden is expressed as a relative number

Italic values indicate significant differences

**Table 3:** Changes of plaque composition in relative and absolute values

	DM (17 pts, 192 segments)	non-DM (44 pts, 506 segments)	p
Fibrous tissue BL %	0.55 ± 0.15	0.55 ± 0.19	0.69
Fibrous tissue FU %	0.57 ± 0.12	0.56 ± 0.19	0.7
Δ Fibrous tissue %	0.03 ± 0.14	0.01 ± 0.14	0.32
p value for change between BL and FU	*0.02*	0.78	
Fibrous tissue BL (mm^2^)	1.80 ± 1.31	2.01 ± 1.41	0.26
Fibrous tissue FU (mm^2^)	2.00 ± 1.51	1.89 ± 1.41	0.92
Δ Fibrous tissue (mm^2^)	0.21 ± 0.75	− 0.12 ± 0.60	*0.001*
p value for change between BL and FU	*0.037*	*0.05*	
Fibrous-fatty tissue BL %	0.15 ± 0.12	0.14 ± 0.11	0.81
Fibrous-fatty tissue FU %	0.15 ± 0.08	0.14 ± 0.11	0.84
Δ Fibrous-fatty tissue %	0.0 ± 0.13	0.0 ± 0.12	0.71
p value for change between BL and FU	0.83	0.42	
Fibrous-fatty tissue BL (mm^2^)	0.41 ± 0.45	0.52 ± 0.55	0.12
Fibrous-fatty tissue FU (mm^2^)	0.56 ± 0.62	0.51 ± 0.58	0.86
Δ Fibrous-fatty tissue (mm^2^)	0.15 ± 0.50	− 0.00 ± 0.49	0.17
p value for change between BL and FU	*0.002*	0.87	
NC BL %	0.18 ± 0.12	0.15 ± 0.15	0.43
NC FU %	0.16 ± 0.07	0.14 ± 0.09	0.17
Δ NC %	− 0.02 ± 0.12	− 0.02 ± 0.1	0.94
p value for change between BL and FU	*0.03*	*0.003*	
NC BL (mm^2^)	0.83 ± 1.02	0.68 ± 0.86	0.66
NC FU (mm^2^)	0.63 ± 0.55	0.53 ± 0.59	0.51
Δ NC (mm^2^)	− 0.20 ± 0.65	−0.15 ± 0.49	0.9
p value for change between BL and FU	*0.001*	*< 0.001*	
NC abutting lumen BL (°)	61.36 ± 58.65	54.32 ± 57.43	0.75
NC abutting lumen FU (°)	62.63 ± 53.89	51.05 ± 51.02	0.26
Δ NC abutting lumen (°)	1.27 ± 50.23	− 3.27 ± 41.79	0.41
p value for change between BL and FU	0.79	0.24	
DC BL %	0.1 ± 0.09	0.08 ± 0.09	0.26
DC FU %	0.09 ± 0.07	0.08 ± 0.08	0.11
Δ DC %	0.0 ± 0.09	0.0 ± 0.07	0.93
p value for change between BL and FU	0.95	0.99	
DC BL (mm^2^)	0.43 ± 0.55	0.33 ± 0.50	0.37
DC FU (mm^2^)	0.37 ± 0.39	0.29 ± 0.40	0.26
Δ DC (mm^2^)	−0.06 ± 0.34	− 0.04 ± 0.24	0.82
p value for change between BL and FU	0.1	0.06	

We found higher LAPS at follow-up in diabetics compared to non-diabetics (0.13 ± 1.89 vs. − 0.38 ± 1.8, p = 0.002). DM patients also experienced an increase of LAPS during the study, unlike non-DM patients for which LAPS decreased (0.13 ± 1.79 vs. − 0.23 ± 1.66, p = 0.018)

Italic values indicate significant differences

**Table 4:** Liverpool active plaque score and its changes

	DM (17 pts.)	Non-DM (44 pts.)	p
Baseline LAPS	0.0 ± 1.87	− 0.15 ± 2.06	0.36
Follow-up LAPS	0.13 ± 1.89	− 0.38 ± 1.8	0.002
Change of LAPS	0.13 ± 1.79	− 0.23 ± 1.66	0.018
p value (BL vs. FU)	0.5	0.06	

**Fig. 3:**
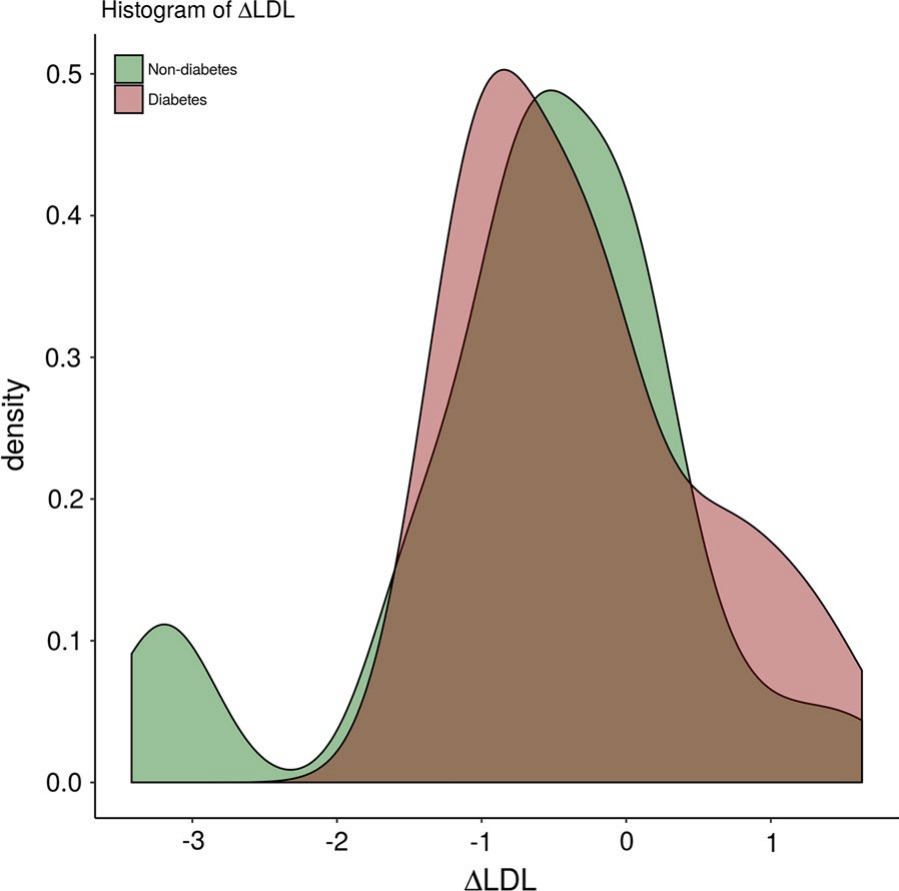
Distribution of LDLc changes in DM and non-DM patients

Despite reaching similar levels of LDL cholesterol compared to non-DM patients (DM 2.12 ± 0.91 mmol/l, non-DM 1.8 ± 0.66 mmol/l, p = 0.21), DM patients experienced progression of both plaque area (0.21 ± 0.97 vs. − 0.47 ± 1.15, p = 0.001) and percent atheroma volume (0.7% ± 2.8% vs. − 1.4% ± 2.5%, p = 0.007). While the change of LDLc from baseline to follow-up was significant in the non-DM group (p < 0.001), it was non-significant in the DM group (p = 0.15). Distribution of LDLc changes is shown in Fig. 3. We found a significant correlation between the change of LDLc and the change of mean plaque area for non-DM patients (R = 0.47, p = 0.002). No significant correlation was found in DM patients.

**Fig. 4:**
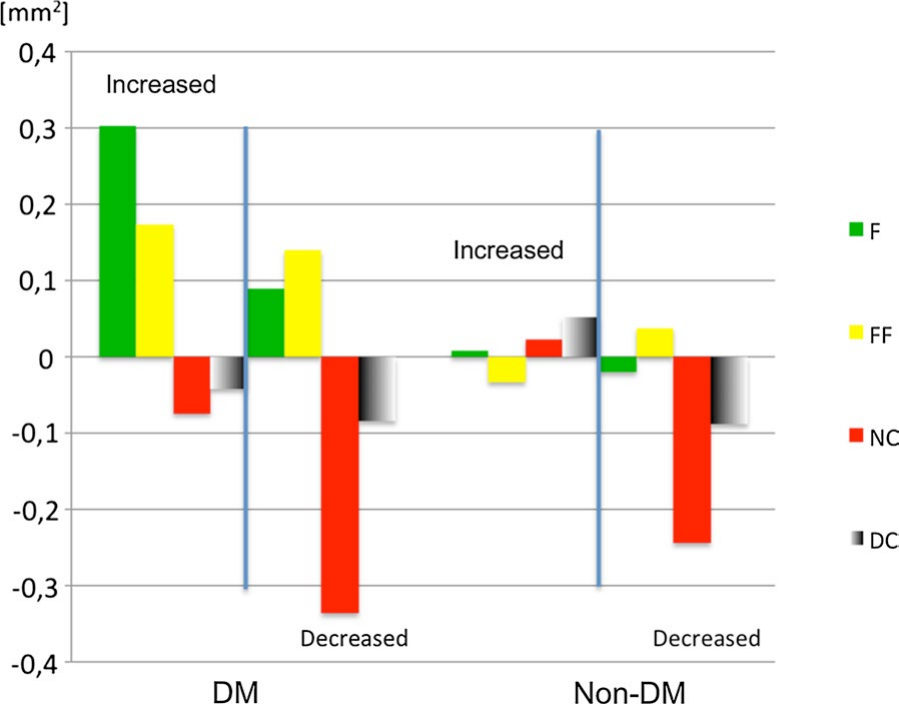
Plaque compositions responsible for either the plaque increase or the plaque decrease. *F* fibrous tissue, *FF* fibro-fatty tissue, *NC* necrotic tissue, *DC* calcified tissue

The only significant difference in plaque composition was an increase of fibrous tissue in DM patients (0.21 ± 0.75 mm^2^) compare to a decrease in non-DM patients (− 0.12 ± 0.60 mm^2^, *p* = 0.001). Interestingly, both NC tissue and DC decreased in both group (differences between DM and non-DM groups were not significant). One of the most indicative markers for plaque composition risk—NC abutting lumen—increased in DM patients (1.27° ± 50.23°) and decreased in non-DM patients (− 3.27° ± 41.79°). However, this difference was not significant (p = 0.47). Plaque components responsible for increase or decrease of plaque area are summarized in Fig. 4. No change of plaque composition was significant when the DM and non-DM groups were compared. Plaque progression in DM patients was caused by an increase of fibrous and fibro-fatty tissue mean area. Progression in non-DM patients was negligible. Plaque regression was caused by decrease of necrotic tissue and calcified tissue mean area in both groups.

Changes of the pro-inflammatory status were related to changes of plaque composition in the DM patients. The change of hs-CRP level correlated well with the increase of DC mean area (R = 0.57, p = 0.03), decrease of fibrous tissue mean area (R = − 0.69, p = 0.01) and there was a trend for increase of NC mean area (R = 0.46, p = 0.09). All these changes were not significant in non-DM patients: correlation coefficients were much smaller (0.22 for NC, 0.21 for DC and − 0.27 for fibrous tissue, with non-significant “p” values). However, the change of hs-CRP level correlated better with change of the TCFA number in non-DM patients (R = 0.36, p = 0.02).

Glycemia levels correlated well in both the baseline (R = 0.55, p = 0.02) and the follow-up plaque burden (R = 0.57, p = 0.02) in DM patients. Glycemia changes correlated well with changes of the necrotic tissue (R = 0.41, p = 0.01) and the fibro-fatty tissue (R = − 0.33, p = 0.04), but only in the non-DM patients. Baseline glycemia or change of glycemia did not correlate with number of TCFA locations during baseline examination or change of TCFA number during the study.

**Fig. 5:**
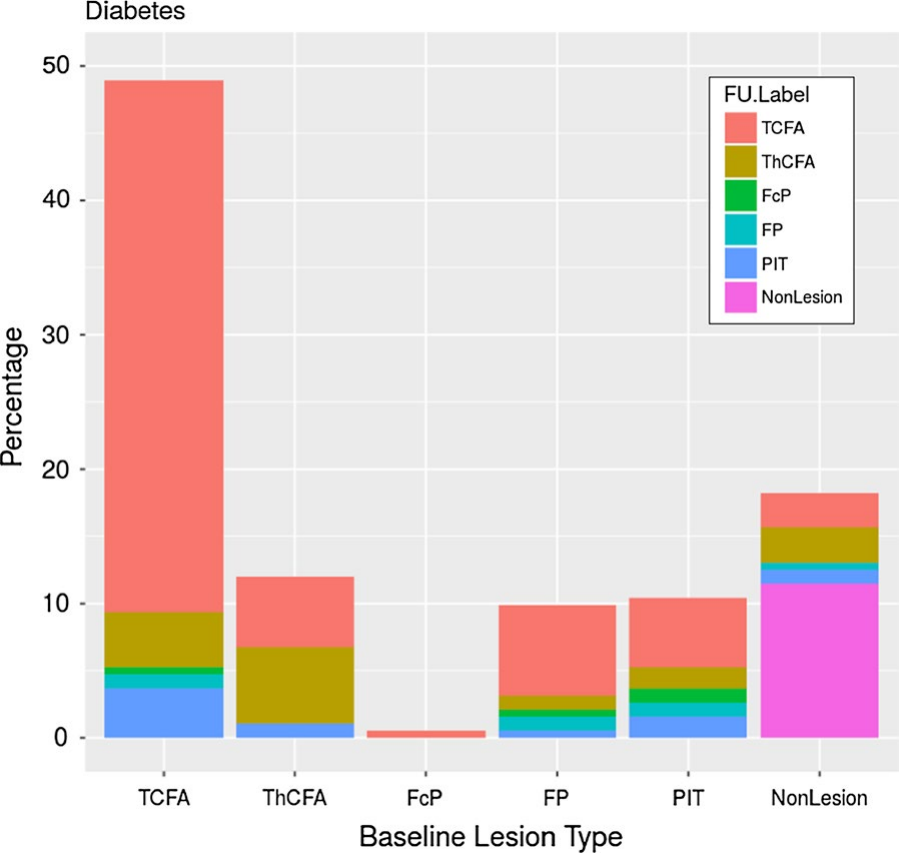
Plaque phenotype transition in DM patients. The “y” axis represents relative occurrence of a plaque phenotype in 5 mm vessel segments during baseline. Changes into follow-up plaque phenotypes are expressed in colors within each baseline column

**Fig. 6:**
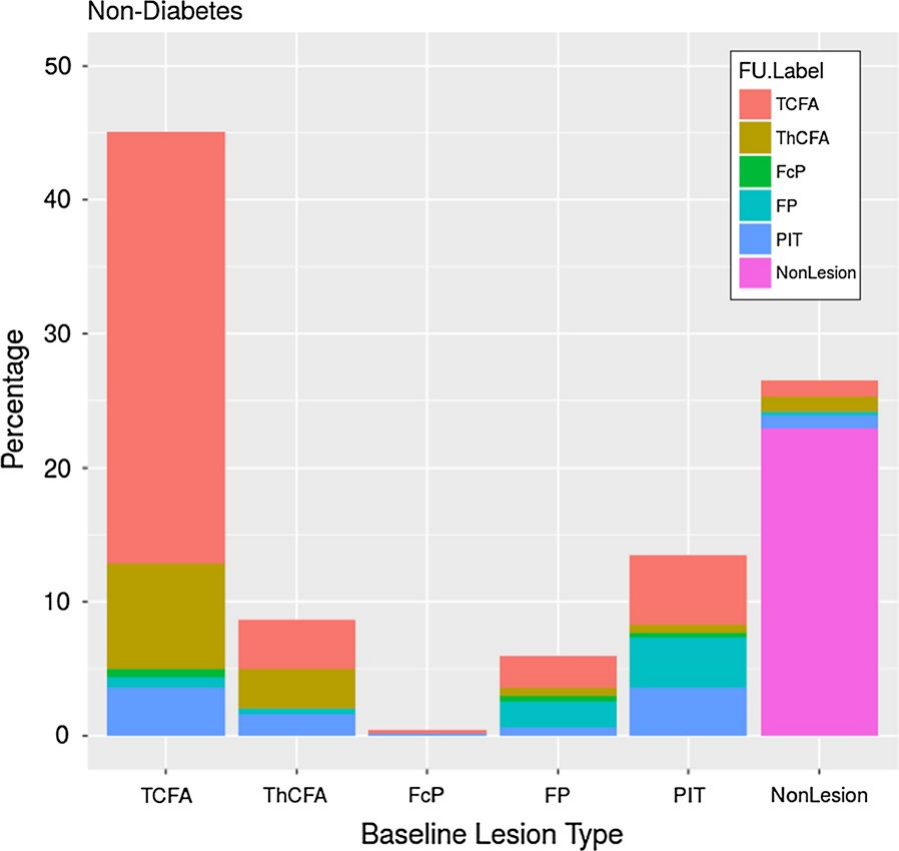
Plaque phenotype transition in non-DM patients. The “y” axis represents relative occurrence of a plaque phenotype in 5 mm vessel segments during baseline. Changes into follow-up plaque phenotype are expressed in colors within each baseline column

**Fig. 7:**
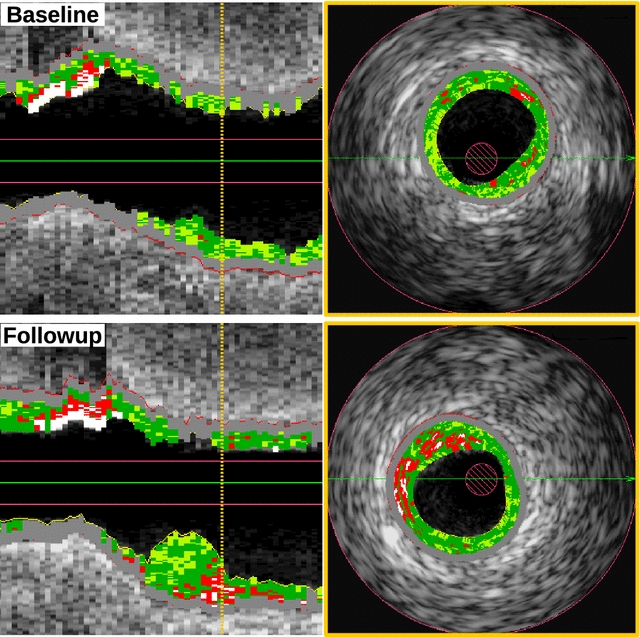
Longitudinal and cross section views of one baseline and follow-up pullbacks showing development of new TCFA in DM patients. Orange line shows location of cross sectional frames. It can been seen significant progression of plaque together with increase of necrotic core

Figures 5 and 6 show the direction of changes in plaque phenotypes between baseline and follow-up in DM and non-DM patients. It can be clearly seen that changes of plaque phenotypes from early lesions (NL, FP, PIT) into advanced plaque phenotypes (ThCFA, TCFA) were more frequent in DM compared to non-DM patients. However, TCFA plaque phenotype was found in 94 of 192 (48.9%) vessel segments in DM patients and in 228 of 506 vessel segments (45.1%) in non-DM patients (p = 0.96), in part because we labeled each vessel segment according to the most advance identifiable plaque phenotype. In fact the total number of “TCFA frames” was lower, but still without a significant difference between the DM and non-DM groups (24.8% vs, 23.4%, p = 0.29). Figure 7 is showing and example of new TCFA in DM patient.

The TCFA plaque phenotype can experience two types of behavior. It can remain as TCFA (*persistent TCFA*), or it can change into another plaque phenotypes (suggesting a *healed TCFA*). An TCFA can also develop from another plaque phenotype during the follow-up period (*new TCFA)*. DM patients experienced more new TCFA plaque phenotype compared to non-DM patients (20.3% vs. 12.6%, p = 0.01). Persistent TCFA plaque phenotype was more frequent in DM patients, but this trend was not statistically significant (82.1% vs. 71.3%, p = 0.12).

We also focused on differences between the DM and non-DM groups in morphological and plaque composition changes in early plaque phenotype (PIT) and advanced plaque phenotype (TCFA). Both PIT and TCFA types of plaques experienced plaque regression together with negative vessel remodeling in non-DM patients and plaque progression together with positive (TCFA) or neutral (PIT) vessel remodeling in DM patients.

**Fig. 8:**
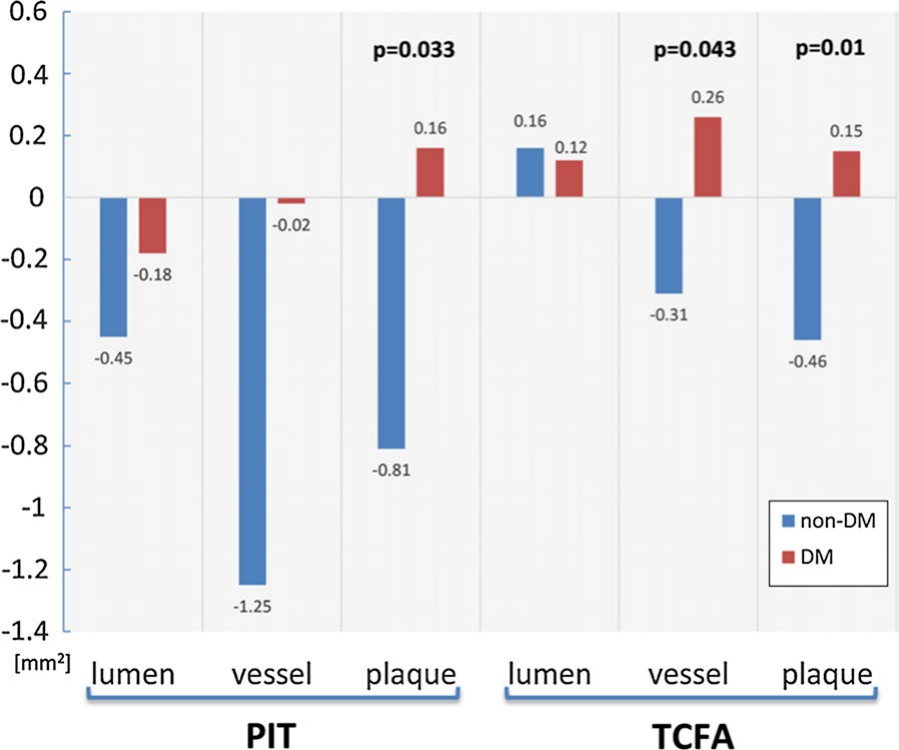
Changes of mean lumen, vessel and plaque area per 5 mm vessel segments in PIT and TCFA phenotypes in DM vs. non-DM patients

**Fig. 9:**
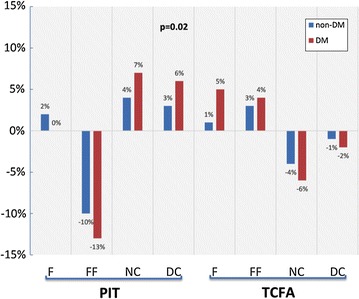
Changes of plaque composition in PIT and TCFA plaque phenotype in DM vs. non-DM patients. Values in “y” axis are expressed as relative amount

**Fig. 10:**
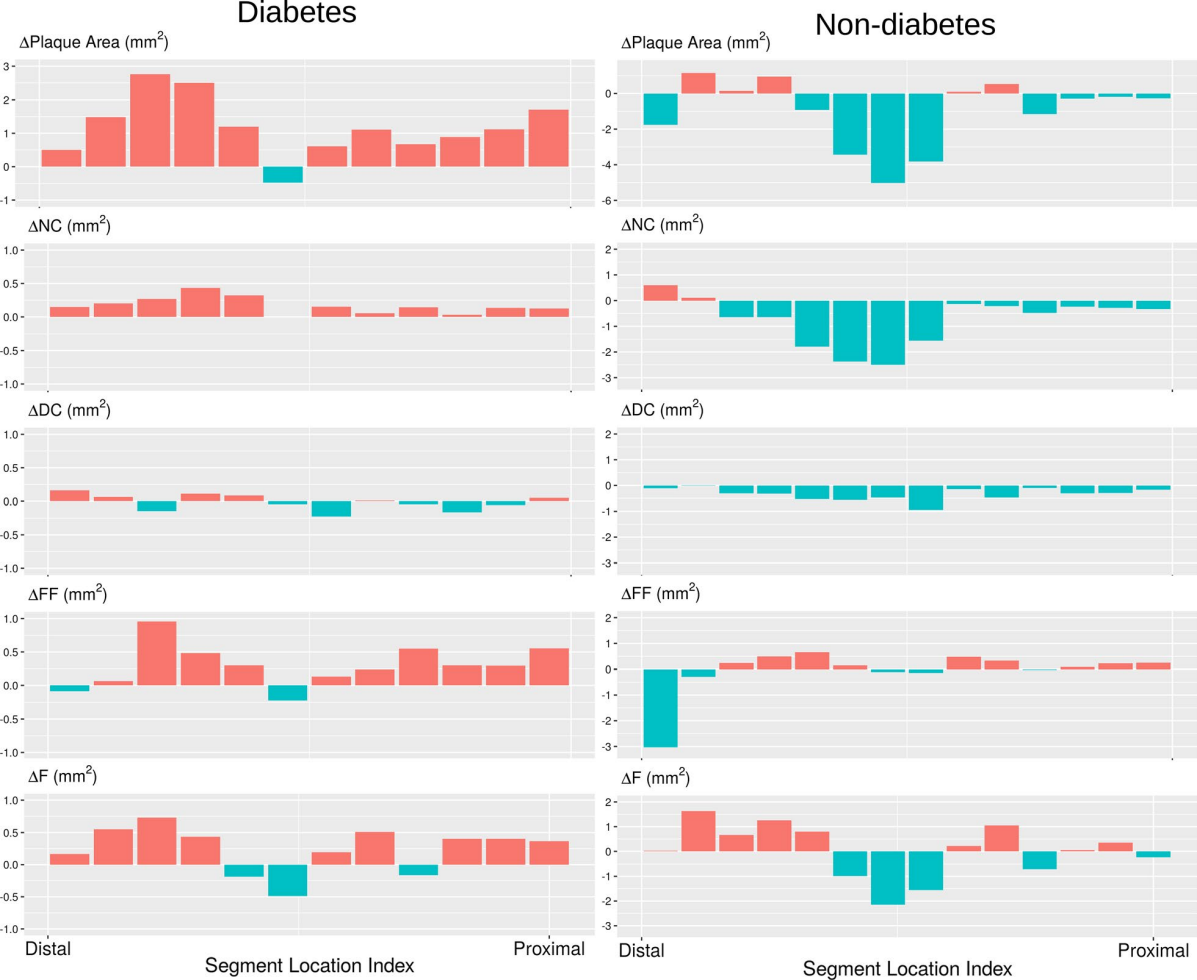
3D analysis of typical DM and non-DM patients differences in plaque area changes and plaque composition changes are clearly visible

We found interesting differences between PIT and TCFA plaque phenotypes according to change of plaque composition. Plaque progression in PIT plaque phenotype was caused by increases of NC and DC plaque composition, but plaque progression in TCFA plaque phenotype was caused by increases of fibrous and fibro-fatty tissue. Results are summarized in Figs. 8 and 9. Figure 10 shows a typical 3D analysis of coronary IVUS pullbacks in DM and non-DM patients. Note the visible differences in plaque area and plaque composition.

## Discussion

The main findings of this study are:

1. The coronary plaques in DM patients increased their plaque area and risk profile from baseline to 1-year follow up despite treatment with lipid lowering therapy and despite reaching a similar level of LDLc compared to non-DM patients. In comparison, plaques in non-DM patients experienced a decrease of the LAPS risk score.
2. The plaque phenotype with the highest risk of future cardiac events (TCFA) developed more frequently during the study in DM patients compared to non-DM patients.
3. Coronary plaques continue to progress in both early and advanced plaque in DM patients compared to plaque regression on these two plaque phenotypes in non-DM patients.
4. Morphological and plaque composition changes were more pronounced in the early lesions type (PIT) than in the advanced lesion type (TCFA), and these changes transitioned toward higher risk plaque types (increased mean plaque area and necrotic core content) in DM patients.

The atherosclerotic process in diabetic patients seems to be different compared to non-diabetics. Larger plaques, with higher necrotic core content, were confirmed in DM patients during postmortem studies [27]. These findings were confirmed in vivo by studies using IVUS in plaques from both stable and acute patients [28–30]. Studies assessing plaque phenotypes describe more-developed lesions in DM patients [31, 32]. We found accelerated progression in DM patients of both plaque burden and plaque risk profiles in our study. These findings are supported by the study published by Bayturan et al. [10] with data from 7 clinical trials involving 3437 patients, where the presence of diabetes was found as one of the predictors of plaque progression despite the achievement of very low levels of LDLc. The impact of the presence of diabetes on clinical events was tested in a study done by Kennedy et al. Lesions not causing ischemia (with fraction flow reserve > 0.8) led to clinical events in DM patients in 18.1% compared to 7.5% in non-DM patients (p < 0.01), with hazard ratio for the presence of DM of 3.3 [33]. The same author published a provocative study where he suggests to routinely perform PCI of FFR negative lesions for poor outcome of such lesions for fast atherosclerosis progression in DM patients [34].

Factors that can play an important role in faster progression in DM patients are: inflammation, neovascularization, and intra-plaque hemorrhage [35]. Neovessels provide access for inflammatory cells, and thus correlate with plaque inflammation [36]. Cytokines coming from leucocytes decrease collagen production by vascular smooth muscle cells, and enhance production of matrix metalloproteinases, which further weaken the plaque stability through fibrous cap breakdown [37]. Metalloproteases are also important factor for development of positive vessel remodeling that is also known risk factor for plaque instability. Insulin resistance was shown as a factor related to positive vessel remodeling [38]. Neovessels are more fragile and therefore more prone to rupture, thus causing intra-plaque hemorrhage [39]. These processes are augmented in the diabetic plaques [35]. These studies give us the pathological background for our finding of the accelerated progression of plaque phenotype in DM patients, which now look more like expected results than merely accidental findings.

These data are in agreement with our findings of plaque area, remodeling index (marker of positive vessel remodeling) and LAPS score progression contributing to disease progression in DM patients. Interestingly, the amount of NC significantly decreased in both groups of patients. Only the amount of NC abutting lumen increased in DM patient and decreased in non-DM ones, but this difference did not reach statistical significance.

Plaque progression and increased necrotic core in DM patients was previously reported in the TRUTH study^4^. Inaba et al. [40] performed a serial IVUS study using integrated back scatter analysis (IB IVUS) for examination of plaque composition. They compared 20 mm of non-culprit coronary artery from DM and non-DM patients and found higher total plaque volume and lipid content in DM patients at baseline. Both plaque volume and lipid content continued to progress only in the DM patients. However, comparison of plaque composition changes is difficult using their approach, because IB-IVUS uses a different technique for analysis of plaque composition and cannot distinguish necrotic tissue [41].

The possible explanations for differences in plaque-type and plaque-composition transition frequencies in DM and non-DM patients during the 12-month follow-up period can be attributed to a lower efficacy of lipid-lowering treatment in DM patients, which was documented by observing a significant reduction of LDLc levels only in the non-DM patients. Another factor contributing to the unfavorable plaque changes is a higher inflammatory status biomarker in DM patients. Levels of hs-CRP during baseline and their changes during the study duration were significantly higher only in the non-DM group. However, the change of hs-CRP levels correlated with an increase of the necrotic core percentage and calcification, and the decrease of fibrous tissue more strongly in the DM patients. These findings are in a good agreement with a study performed by Kwon et al. [42], who found a decrease of necrotic tissue inside coronary plaque in statin-treated patients with decreased levels of hs-CRP. This can also be seen as an indirect marker of the more important role of inflammation in DM patients compared to the non DM ones. Surprisingly, the increase of TCFA plaque phenotype correlated with increase of hs-CRP level only in the non-DM patients, despite the fact that the DM patients developed more new TCFA plaque phenotypes.

Additional differences between the DM and non-DM patients included a higher glucose variability in DM patients leading to an increase of plaque burden and lipid content and a decrease of fibrous tissue in their atherosclerotic plaques [43]. Yoshida et al. [44] described an increase of necrotic core in plaque with DM patients with higher glucose fluctuations. We found a notable correlation of both baseline and follow-up glycemia with plaque burden in the DM patients. According to plaque composition, we found good correlation of he change of glycemia with the decrease of fibro-fatty tissue and the increase of necrotic tissue. Surprisingly these correlations were significant only in the non-DM patients (probably due to a negligible change of glycemia in the DM patients). These findings may explain the reported relationships between an increase of hs-CRP and an increase of TCFA plaques in the non-DM patients.

TCFA is a plaque phenotype, defined as a confluent NC with a thin fibrous cap (less than 65 µm). Because this distance cannot be measured by intravascular ultrasound, TCFA plaque phenotype is sometimes named VH-TCFA (TCFA based on virtual histology) or ID-TCFA (IVUS-derived TCFA) in studies using virtual histology, where the main criterion for this phenotype is the lack of a visible fibrous cap over the necrotic core. This type of plaque is known as a risk factor for future development of coronary events [45]. We find similar numbers of such plaque phenotypes in both patients’ groups. The same occurrence of TCFA in DM and non-DM patients is a surprising finding. But it is similar to a study done by Pundziute et al. [46], who did not find a higher occurrence of TCFA in DM patients. However, they found a substantially smaller number of TCFA type plaques (7% in DM and 10% in non-DM patients, p = 0.4), but they reported number of TCFAs per whole plaque, not per vessel segment or frame. Other trials [31, 47] found TCFA more frequent in DM patients (21.6% vs. 13.6% and 75% vs. 41%). Based on these numbers, it is obvious that TCFA definition based on IVUS-VH yields different values the absolute numbers of which are generally incomparable.

Because the determining part of the TCFA definition is fibrous cap thickness, studies using optical coherence tomography (OCT) seem to offer a better tool for its diagnosis. Kato et al. [48] published a study of OCT examinations of all three coronary arteries, and found only non-significantly higher numbers of TCFA plaque phenotype in DM patients than in non-DM ones (18.8% vs. 11%, p = 0.22). Niccoli [49] found the same number of TCFA in DM and non-DM patients (41% vs. 44%, p = 1). It seems that even OCT based TCFA definition is not ideal for discrimination of risk plaque phenotype in DM patients. The best approach may be a dual source of information composed of IVUS-VH for necrotic core detection and OCT for fibrous cap measurement [50] or even better near-infrared spectroscopy IVUS (for highly sensitive lipid pool detection) and OCT [51].

The behavior of TCFA over the period of 1 year has been studied by IVUS-VH in patients with stable coronary artery disease [52], and using non-culprit plaques in patients with acute myocardial infarction [53]. During these studies (over a period of 12 months in Kubo et al. [52] and 13 months in Zhiao et al. [53]), 75% of VH-TCFA healed, whereas 25% remained unchanged in stable patients. Completely different results were found in acute patients: 78% of VH-TCFA plaques remained unchanged and 22% healed. We found an increased number of new TCFA plaque phenotypes during our study. From all TCFA plaque types found during baseline examination, 82.1% remained as TCFA in DM patients and 71.3%, (p = 0.12) in non-DM ones, despite dealing with stable patients only. The main difference between our study and the aforementioned studies is lesion definition. Kubo [52] defined lesions as an area of plaque with at least three consecutive frames with plaque burden ≥ 40%, and analyzed all such frames as one lesion. The same definition was used in the study by Zhiao [53] (where new lesions were separated by at least a 5 mm long segment with plaque burden < 40%). Unlike these two studies, we analyzed vessel segments of coronary arteries of uniform size (5 mm). This method allows more precise spatial accuracy for comparison from baseline to follow up. Acute cardiac events are not caused by changes taking place inside the whole plaque: the development of plaque rupture is a very focal event, and only detailed plaque analysis can help us understand why some regions behave differently than others.

Vessel segments with new TCFA plaque phenotype were found in higher numbers in DM compared to non-DM patients in our study. This finding of higher incidence of new TCFA in DM patients despite lipid-lowering treatment correlates well with the study done by Lindsey et al. [54], who also reported correlation between prevalence of TCFA lesions and duration of diabetes.

This is important, because DM patients with TCFA have higher occurrence of MACE within 3 years, compared to DM ones without TCFA. Plaque progression in DM patients without TCFA led to similar occurrence of MACE like in non-DM patients [55].

An interesting finding is the different plaque behavior in early plaque phenotype (PIT) and advanced plaque phenotype (TCFA) in DM patients compared to non-DM patients. Changes in plaque volume and plaque composition are more pronounced in PIT than in TCFA. Plaque regression was found only in non-DM patients in both plaque phenotypes. The relative amount of necrotic core in plaque between baseline and follow-up increased in the PIT phenotype but decreased in the TCFA plaque phenotype. A similar behavior of NC was described by Hwang et al. [56], who found an increase of NC in non-TCFA plaques and a decrease in TCFA plaques during statin therapy in patients with acute coronary syndrome. Increase of NC closely correlated with changes of hs-CRP in that study. Unfortunately, we do not have data from our patients for this type of correlation. It seems that early lesion phenotype is much more active and it may be the main precursor of fibroatheromas despite statin therapy.

## Conclusions

Atherosclerotic plaques in DM patients have more advanced risk profiles than plaques form non-DM patients, and these differences continue to progress despite lipid lowering therapy and despite reaching similar LDLc levels in both groups of patients. In contrary to the group of non-DM patients, the DM patients did not reach significant reduction of LDLc, which shows a decreased efficacy of the lipid lowering treatment in the DM patients. This finding can explain some differences that were observed in our study. The TCFA plaque phenotype detected by virtual histology is probably not the best discriminator for detection of high-risk plaques, because it was found in both groups of patients with the same frequency.

Changes in a plaque morphology and plaque composition are more pronounced in early types of lesions such as PIT. Our findings result from a novel method for detailed analysis of coronary arteries, which divides plaques into 5 mm vessel segments based on 3-D vessel reconstructions. This was done by fusion of IVUS-VH and angiography. This type of analysis allows us to compare corresponding vessel segments of coronary arteries between baseline and follow-up, and to focus on changes of plaque phenotypes, which play a critical role in the development of acute coronary events.

These findings should lead to further examination of plaque progression in DM patients and to development of new therapeutic strategies, which would increase the efficacy of lowering LDLc and slow the progression of atherosclerosis in DM patients. First recommendations come from the IMPROVE IT trial [57], where a combined lipid lowering therapy (statin + ezetimibe) was more efficient in preventing cardiac events in DM patients then in non-DM ones. The most potent lipid-lowering drug (inhibitors of the PCSK-9 protein) was shown as potentially causing a higher plaque regression in DM patients in GLAGOV trial. However this finding did not reach statistical significance (p = 0.39) in a trial with only 20% occurrence of DM patients [58].

## Limitations

The main limitation of our study was the low number of patients, which was the result of strict inclusion criteria for high image quality of both baseline and follow-up IVUS examinations, with reliable and consistent pullbacks, and with unquestionably identified corresponding frames. On the other hand, the comparison of almost 600 corresponding 5 mm vessel segments of coronary arteries compensates for this disadvantage. A further limitation is the unequal number of patients with and without diabetes (17 vs. 44 patients). However, the roughly 38% presence of diabetes in our group corresponds well with the occurrence of diabetic patients among individuals with established coronary artery disease.

According to our results, the TCFA plaque phenotype was the most frequent plaque phenotype found in both DM and non-DM patients. This finding is a result of our TCFA definition. We divided all examined segment of each coronary artery into 5 mm segments that were labeled according to the worst plaque phenotype they contained. So, every time TCFA was part of 5 mm examined vessel this segment was labeled as TCFA. Moreover, it is necessary to find three consecutive frames with TCFA features to satisfy our TCFA definition. In situations where those three frames belonged into two different 5-mm vessel sections, both were labeled as “TCFA”. One of the most important parts of TCFA definition is the required presence of a thin fibrous cap. It means fibrous cap thinner than 65 μm. This value is below the resolution of intravascular ultrasound and therefore cannot be used in an IVUS-based trial. These are all facts, which may lead to an overestimation of the number of identified locations with the TCFA plaque phenotype. However, our main goal was to compare plaque phenotypes present in DM and non-DM patients using the same approach for both groups. Based on our findings, our fundamental results are in general agreement with similar studies, despite using slightly different methods and our detailed analysis brings new knowledge about local behavior of DM and non-DM plaque transitions.

## Abbreviations

ACEI: angiotensin converting enzyme
BL: baseline
CABG: coronary artery bypass graft
CAG: coronary angiography
CSA: cross sectional area
DC: dense calcification
DM: diabetes mellitus
EEM: external elastic membrane
F: fibrous tissue
FcP: fibro-calcific plaque phenotype
FF: fibro-fatty tissue
FP: fibrous plaque phenotype
FU: follow up
HDLc: high-density lipoprotein cholesterol
hs-CRP: high sensitivity C reactive protein
Ch: cholesterol
IB-IVUS: integrated backscatter
IVUS: intravascular ultrasound
IVUS-VH: virtual histology
MACE: major cardiac adverse event
MHz: mega Hertz
MI: myocardial infarction
MLA: minimal lumen area
NC: necrotic core
NIH: National Institute of Health
LAPS: liverpool active plaque score
LDLc: low density lipoprotein cholesterol
NL: no lesion plaque phenotype
Non-DM: not diabetes mellitus
OCT: optical coherence tomography
PCI: percutaneous coronary intervention
PIT: pathological intimal thickening plaque phenotype
TAG: triacyl-glycerol
TCFA: thin cap fibroatheroma plaque phenotype
ThCFA: thick cap fibroatheroma plaque phenotype
TK: Tomas Kovarnik
USA: United States of America
